# Supplementary material for: Nutrient uplift in a cyclonic eddy increases diversity, primary productivity and iron demand of microbial communities relative to a western boundary current
Source: PeerJ. 2016 Apr 25;4:e1973. doi: 10.7717/peerj.1973 (PMC4860325; doi:10.7717/peerj.1973)
Supplement: Supplemental Information 1 [file peerj-04-1973-s001.docx]

**Nutrient uplift in a cyclonic eddy increases diversity, primary productivity and iron demand of microbial communities relative to a western boundary current**

Doblin et al.

**Supplementary Figures**

Figure S1. Initial abundance of major phototrophs in the EAC and CCE microbial communities.

Figure S2. Rates of macronutrient uptake by microbial communities in the East Australian Current (EAC) and cyclonic cold-core eddy (CCE) under different nutrient amdendment. Con = unamended control, +N = nitrate (10 μM), +NFe = nitrate+iron (10 μM and 1nM, respectively), +Si = silicate (10 μM), +Mix = nitrate + phosphate + silicate + iron (10 μM, 0.625 μM, 10 μM, 1nM, respectively).


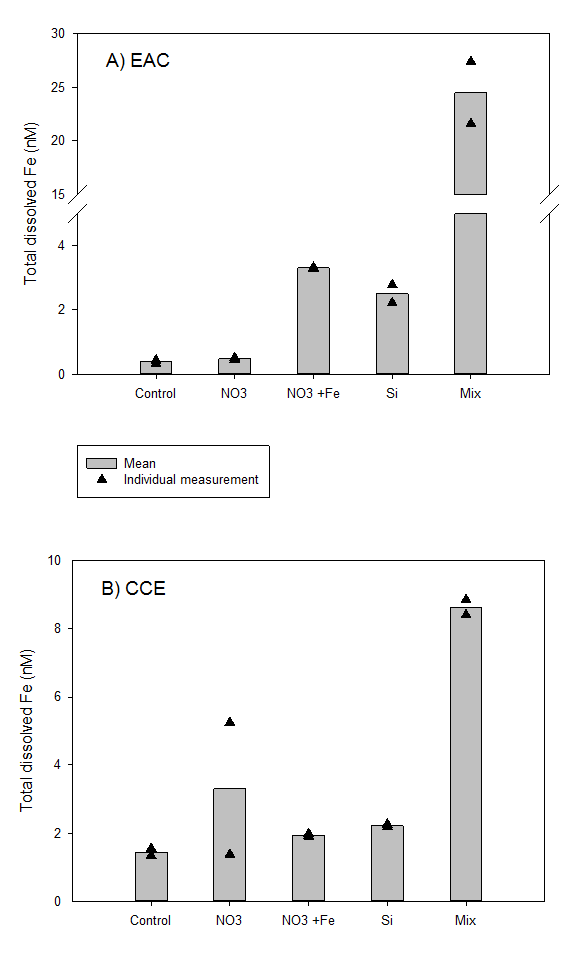


Figure S3. Concentration of total dissolved iron in experimental bottles at the end of the experiment. Values are means of two replicates.

**Supplementary tables:**

Table S1: Primers used for microbial analyses.

| Target | Gene | Forward primer | Reverse primer | Reference |
| --- | --- | --- | --- | --- |
| Bacteria | 16S rRNA | 5’- AGRGTTTGATCMTGGCTCAG-3’ | 5’- GWATTACCGCGGCKGCTG-3’ | Lane 1991 |
| Diazotrophs | nifH | 5'-TTYTAYGGNAARGGNGG-3' (#3)  Followed by  5'-TGYGAYCCNAARGCNGA-3' (#1) | 5'-ATRTTRTTNGCNGCRTA-3' (#4)  Followed by  5'-ADNGCCATCATYTCNCC-3' (#2) | Zehr et al. 1997, 2003 |

Table S2: Mean±SE of parameters at the start and end of the experiment (t_0_ and t_72_, respectively) in each treatment in the East Australian Current (EAC) and cyclonic cold-core eddy (CCE). *Note that the Fe treatment inadvertently contained NO_3_ so we report values for NO_3_ and NO_3_+Fe treatments in the main text.

|  | T_0_ | | Control | | NO_3_ | | Fe* | | NO_3_+Fe | | Si | | Mix | |
| --- | --- | --- | --- | --- | --- | --- | --- | --- | --- | --- | --- | --- | --- | --- |
|  | EAC | CCE | EAC | CCE | EAC | CCE | EAC | CCE | EAC | CCE | EAC | CCE | EAC | CCE |
| Total PE fluorescence/cell (x10^4^) | 967±  20 | 1440±  34 | 916±  18 | 1320±  42 | 1564± 11 | 1679±  17 | 1497±  3 | 1573±  5 | 1440±  14 | 1594±  24 | 1001±  7 | 1484±  25 | 1520±  15 | 1769±  32 |
| Prochlorococcus PE fluorescence/cell (x10^6^) | 335±2 | 707±10 | 524±7 | 683±14 | 548±2 | 677±4 | 556±21 | 672±2 | 523±5 | 718±9 | 535±4 | 566±10 | 583±4 | 543±20 |
| Synechococcus PE fluorescence/cell (x10^4^) | 898±  79 | 1211±  130 | 848±  71 | 1104±  146 | 1492±  44 | 1476±  65 | 1429±  6 | 1390±  14 | 1377±  52 | 1449±  85 | 930±  26 | 1404±  97 | 1439±  55 | 1699±  124 |
| Picoeukaryote PE fluorescence/cell (x10^6^) | 1019±  23 | 1196±  36 | 1218±  29 | 1105±  42 | 1460±  26 | 1227±  41 | 1505±  31 | 1279±  26 | 1371±  84 | 1150±  89 | 1319±  17 | 952±  29 | 1488±  72 | 957±  10 |
| Nanoeukaryote PE fluorescence/cell (x10^4^) | 56±2 | 210±6 | 51±2 | 198±21 | 52±1 | 186±4 | 47±4 | 163±7 | 43±1 | 126±9 | 53±3 | 65±1 | 61±2 | 55±3 |
| Total Chl  *a*  fluorescence/cell | 29.89± 0.34 | 44.28+ 0.34 | 16.79± 0.32 | 42.71± 0.42 | 24.10± 0.22 | 50.54+ 0.17 | 23.44± 0.31 | 47.28+ 0.15 | 20.94± 0.13 | 34.03± 0.33 | 17.14± 0.07 | 25.06± 0.35 | 23.86± 0.21 | 24.55± 0.22 |
| Prochlorococcus Chl  *a* fluorescence/cell | 0.066± 0.001 | 0.042± 0.001 | 0.059± 0.006 | 0.044± 0.003 | 0.064± 0.001 | 0.041± 0.001 | 0.066± 0.002 | 0.045± 0.002 | 0.062± 0.001 | 0.042± 0.001 | 0.053± 0.004 | 0.046± 0.002 | 0.059± 0.003 | 0.051± 0.001 |
| Synechococcus Chl  *a*  fluorescence/cell | 0.41± 0.01 | 0.51± 0.05 | 0.45± 0.04 | 0.50± 0.07 | 0.79± 0.02 | 0.68± 0.02 | 0.80± 0.02 | 0.65± 0.01 | 0.77± 0.04 | 0.63± 0.05 | 0.54± 0.01 | 0.81± 0.07 | 0.80± 0.02 | 0.90± 0.04 |
| Picoeukaryote Chl  *a*  fluorescence/cell | 3.95± 0.02 | 4.15± 0.19 | 4.00± 0.25 | 3.52± 0.32 | 6.49± 0.20 | 5.51± 0.25 | 5.67± 0.13 | 5.14± 0.17 | 6.11± 0.24 | 4.96± 0.14 | 4.00± 0.06 | 3.67± 0.26 | 4.78± 0.29 | 4.76± 0.24 |
| Nanoeukaryote Chl  *a*  fluorescence/cell | 25.5± 1.3 | 39.6± 1.1 | 12.3± 1.0 | 38.7± 1.3 | 16.8± 0.7 | 44.3± 0.4 | 17.0± 1.1 | 41.5± 0.4 | 14.0± 0.2 | 28.4± 1.2 | 12.5± 0.2 | 20.5± 1.1 | 18.2± 0.5 | 18.8± 0.6 |
| F_V_/F_M_ | 0.701±0.004 | 0.690±0.015 | 0.710±0.008 | 0.637±0.012 | 0.647±0.012 | 0.696±0.020 | 0.664±0.004 | 0.671±0.010 | 0.671±0.010 | 0.695±0.015 | 0.716±0.021 | 0.696±0.008 | 0.681±0.006 | 0.689±0.008 |
| Chl *a* | 0.106± 0.004 | 0.336±0.023 | 0.086±0.014 | 0.205±0.017 | 0.238±0.019 | 0.587±0.096 | 0.228±0.014 | 0.610±0.036 | 0.229±0.022 | 0.698±0.122 | 0.107±0.010 | 0.324±0.018 | 0.245±0.019 | 0.788±0.142 |
| But-Fuco:Chl  *a* | 0.104±0.003 | 0.044±0.001 | 0.102±0.010 | 0.033±0.001 | 0.094±0.003 | 0.023±0.004 | 0.095±0.005 | 0.028±0.002 | 0.101±0.004 | 0.028±0.002 | 0.089±0.006 | 0.038±0.004 | 0.121±0.013 | 0.023±0.001 |
| Fuco:Chl  *a* | 0.150±0.001 | 0.306±0.010 | 0.251±0.020 | 0.297±0.004 | 0.229±0.013 | 0.344±0.010 | 0.263±0.012 | 0.329±0.006 | 0.365±0.013 | 0.365±0.012 | 0.237±0.025 | 0.320±0.005 | 0.339±0.039 | 0.363±0.005 |
| Hex-Fuco:Chl  *a* | 0.492±0.010 | 0.173±0.008 | 0.317±0.030 | 0.220±0.020 | 0.432±0.005 | 0.139±0.013 | 0.400±0.009 | 0.145±0.002 | 0.179±0.009 | 0.108±0.007 | 0.357±0.061 | 0.170±0.006 | 0.274±0.054 | 0.123±0.010 |
| Peridinin:Chl  *a* | 0.043±0.003 | 0.030±0.002 | - | 0.024±0.001 | - | 0.010±0.002 | - | 0.014±0.002 | - | 0.012±0.003 | - | 0.021±0.004 | - | 0.010±0.002 |
| Pmax | 1.56±  0.00 | 0.98±  0.00 | 3.12±  0.20 | 1.11±  0.09 | 5.41±  0.37 | 3.05±  0.30 |  |  | 1.29±  0.05 | 6.99±  0.37 | - | - | - | - |
| alpha | 0.038±  0.000 | 0.007±  0.000 | 0.035± 0.007 | 0.008± 0.001 | 0.056± 0.011 | 0.023± 0.002 |  |  | 0.028± 0.001 | 0.055± 0.004 | - | - | - | - |
| I_k_ | 41±0 | 139±0 | 94±12 | 142±2 | 102±12 | 135±1 |  |  | 46±1 | 128±4 | - | - | - | - |
| Total cell abundance  (cells ml^-1^) | 95996± 570 | 1427± 205 | 60383± 1500 | 32006± 884 | 46307± 252 | 28603± 921 | 45489± 534 | 29943± 610 | 57006± 1252 | 30138± 839 | 48295± 617 | 24205± 1350 | 42836± 654 | 28109± 1040 |
| Prochlorococcus cell abundance (cells ml^-1^) | 74629± 1867 | 3109± 585 | 34000± 3337 | 5730± 430 | 29748± 397 | 7141± 1085 | 29039± 953 | 7354± 1448 | 32003± 1857 | 8918± 1210 | 30874± 1709 | 1622± 813 | 27320± 1567 | 2404± 376 |
| Synechococcus cell abundance (cells ml^-1^) | 16961±  140 | 7463± 105 | 22897± 2095 | 20115± 2758 | 14593± 501 | 14768± 1731 | 14732± 1025 | 1564± 816 | 21247± 2574 | 13485± 1341 | 15753± 619 | 15665± 3873 | 13953± 902 | 18059± 3100 |
| Picoeukaryote cell abundance (cells ml^-1^) | 4029± 223 | 3633± 110 | 3034± 508 | 5834± 314 | 1482± 53 | 6015± 742 | 1317± 125 | 6258± 113 | 2851± 368 | 5923± 653 | 1355± 80 | 6441± 611 | 1039± 109 | 6929± 549 |
| Nanoeukaryote cell abundance (cells ml^-1^) | 378±  49 | 323±  21 | 452±  61 | 327±  36 | 484±  57 | 678±  126 | 402±  35 | 696±  62 | 905±  210 | 812±  151 | 312±  60 | 477±  102 | 523±  37 | 720±  133 |
| Total phototrophs growth rate (d^-1^) | - | - | -0.055±  0.043 | 0.171±  0.032 | -0.153±  0.016 | 0.223±  0.050 | -0.180±  0.025 | 0.262±  0.030 | -0.016±  0.049 | 0.262±  0.043 | -0.190±  0.028 | 0.118±  0.056 | -0.188±  0.021 | 0.165±  0.039 |
| Prochlorococcus growth rate (d^-1^) | - | - | -0.265± 0.031 | 0.202± 0.026 | -0.307± 0.004 | 0.269± 0.051 | -0.315± 0.011 | 0.273± 0.068 | -0.283± 0.049 | 0.345± 0.047 | -0.295± 0.028 | -0.055± 0.029 | -0.336± 0.021 | -0.095± 0.058 |
| Synechococcus growth rate (d^-1^) | - | - | 0.097± 0.031 | 0.324± 0.047 | -0.051± 0.012 | 0.222± 0.042 | -0.049± 0.024 | 0.246± 0.017 | 0.070± 0.041 | 0.194± 0.035 | -0.025± 0.013 | 0.227± 0.082 | -0.066± 0.021 | 0.285± 0.054 |
| Picoeukaryote growth rate (d^-1^) | - | - | -0.105± 0.059 | 0.157± 0.019 | -0.334± 0.012 | 0.163± 0.044 | -0.376± 0.033 | 0.181± 0.006 | -0.121± 0.042 | 0.212± 0.033 | -0.364± 0.020 | 0.188± 0.033 | -0.455± 0.034 | 0.213± 0.027 |
| Nanoeukaryote growth rate (d^-1^) | - | - | 0.053± 0.049 | 0.001± 0.035 | 0.078± 0.037 | 0.236+ 0.064 | 0.018± 0.030 | 0.254± 0.030 | 0.269± 0.091 | 0.297± 0.059 | -0.075± 0.060 | 0.112± 0.080 | 0.107± 0.023 | 0.257± 0.059 |
